# Mapping QTLs using a novel source of salinity tolerance from Hasawi and their interaction with environments in rice

**DOI:** 10.1186/s12284-017-0186-x

**Published:** 2017-11-02

**Authors:** M. Akhlasur Rahman, Isaac Kofi Bimpong, J. B. Bizimana, Evangeline D. Pascual, Marydee Arceta, B. P. Mallikarjuna Swamy, Faty Diaw, M. Sazzadur Rahman, R. K. Singh

**Affiliations:** 1 International Rice Research Institute (IRRI), DAPO Box 7777, Metro Manila, Philippines; 2 Bangladesh Rice Research Institute, Gazipur, 1701 Bangladesh; 3 Africa Rice Center, Sahel Regional Station, BP 96 St Louis, Senegal; 4 IRRI-ESA Office, Bujumbura, Burundi; 5 Institute of Biological Sciences, University of the Philippines at Los Baños, Laguna, Philippines

**Keywords:** Hasawi-*aus* rice landrace, Single nucleotide polymorphism (SNP), Novel QTLs, QTL × environment interactions, Seedling-stage salinity tolerance

## Abstract

**Background:**

Salinity is one of the most severe and widespread abiotic stresses that affect rice production. The identification of major-effect quantitative trait loci (QTLs) for traits related to salinity tolerance and understanding of QTL × environment interactions (QEIs) can help in more precise and faster development of salinity-tolerant rice varieties through marker-assisted breeding. Recombinant inbred lines (RILs) derived from IR29/Hasawi (a novel source of salinity) were screened for salinity tolerance in the IRRI phytotron in the Philippines (E1) and in two other diverse environments in Senegal (E2) and Tanzania (E3). QTLs were mapped for traits related to salinity tolerance at the seedling stage.

**Results:**

The RILs were genotyped using 194 polymorphic SNPs (single nucleotide polymorphisms). After removing segregation distortion markers (SDM), a total of 145 and 135 SNPs were used to construct a genetic linkage map with a length of 1655 and 1662 cM, with an average marker density of 11.4 cM in E1 and 12.3 cM in E2 and E3, respectively. A total of 34 QTLs were identified on 10 chromosomes for five traits using ICIM-ADD and segregation distortion locus (SDL) mapping (IM-ADD) under salinity stress across environments. Eight major genomic regions on chromosome 1 between 170 and 175 cM (*qSES1.3, qSES1.4, qSL1.2, qSL1.3, qRL1.1, qRL1.2, qFWsht1.2, qDWsht1.2*), chromosome 4 at 32 cM (*qSES4.1*, *qFWsht4.2, qDWsht4.2*), chromosome 6 at 115 cM (*qFWsht6.1, qDWsht6.1*), chromosome 8 at 105 cM (*qFWsht8.1, qDWsht8.1*), and chromosome 12 at 78 cM (*qFWsht12.1, qDWsht12.1*) have co-localized QTLs for the multiple traits that might be governing seedling stage salinity tolerance through multiple traits in different phenotyping environments, thus suggesting these as hot spots for tolerance of salinity. Forty-nine and 30 significant pair-wise epistatic interactions were detected between QTL-linked and QTL-unlinked regions using single-environment and multi-environment analyses.

**Conclusions:**

The identification of genomic regions for salinity tolerance in the RILs showed that Hasawi possesses alleles that are novel for salinity tolerance. The common regions for the multiple QTLs across environments as co-localized regions on chromosomes 1, 4, 6, 8, and 12 could be due to linkage or pleiotropic effect, which might be helpful for multiple QTL introgression for marker-assisted breeding programs to improve the salinity tolerance of adaptive and popular but otherwise salinity-sensitive rice varieties.

**Electronic supplementary material:**

The online version of this article (10.1186/s12284-017-0186-x) contains supplementary material, which is available to authorized users.

## Background

Unfavorable environmental conditions such as salinity, drought, heat, and submergence pose a huge threat to agricultural production and productivity and challenge future food security. Abiotic stresses cause crop yield losses of more than 50% and this is expected to worsen further because of climate change, so there is an urgent need to develop climate-smart crop varieties to counteract abiotic stresses and to sustain food production (Zeigler and Barclay 2008; Kumari et al. 2009; Khan et al. 2016). Salinity is one of the widest-spread and most severe abiotic stresses that affect rice production and productivity worldwide (Flowers 2004).

Rice (*Oryza sativa* L.) is the major staple food for almost one-half of the world’s population, so sustained rice production and productivity are essential for food security. However, the rice crop is sensitive to salinity stress during different stages of its growth and development, and this stress at the seedling stage is most severe and can sometimes cause complete crop failure (Munns and Tester 2008; Singh et al. 2010; Hossain et al. 2015; Munns and Gilliham 2015). The prevalence of higher sodium ions (Na^+^) in saline conditions is harmful to the growth and development of rice plants because of the negative effect on photosynthesis that leads to a reduction in plant growth, chlorophyll content, and metabolic processes (Qados 2011; Munns and Gilliham 2015; Rahman et al. 2016). Transplanting cost is one of the major resource-consuming activities and it could be reduced using recent techniques such as direct-seeded rice (DSR). But, DSR is not feasible in salt-affected areas as rice seedlings are very sensitive to salinity stress; hence, the recommendation for salt-affected soils is to plant seedlings older than the normal 21-day-old seedlings. Thus, salinity tolerance at the seedling stage is crucial for good crop establishment, especially in coastal areas. Various mechanisms such as preferential uptake of potassium ions (K^+^), sodium exclusion from roots, and its restricted transport to shoots have been reported to confer salinity tolerance in rice (Kader et al. 2006; Wu et al. 2009; Singh and Flowers 2010; Rahman et al. 2016). However, salinity tolerance is a complex trait governed by genetic factors such as multiple QTLs and their interactions (epistasis), and is also significantly influenced by environmental factors (Wurschum et al. 2013; Roy et al. 2014; Khan et al. 2016; Liu et al. 2016).

Recent advances in molecular marker technology have enabled the dissection of the genetic basis of salinity tolerance to identify major-effect QTLs and their use in marker-assisted breeding to develop salinity-tolerant rice varieties (Munns 2005; Tuberosa and Salvi 2006; Passioura et al. 2007; Thomson et al. 2010, 2012; Thomson 2014; Hossain et al. 2015). Several studies have reported QTLs for traits related to salinity tolerance.

Precise and rapid exploitation of rice germplasm by identifying useful alleles and introgressing them into elite rice varieties is a key to successful breeding programs (Negrao et al. 2008). A new source of salinity tolerance, “Hasawi,” an *aus* landrace from Saudi Arabia, is found to have higher Na^+^ exclusion and early seedling vigor (Thomson et al. 2010; Al-Mssallem et al. 2011; Rahman et al. 2016; Bizimana et al. 2017). Salinity tolerance in Pokkali is due to its capacity to maintain a low Na^+^-K^+^ ratio in the shoot tissue (ion-homeostasis) and its faster growth rate under saline conditions (Walia et al. 2005; Ismail et al. 2007; Singh et al. 2010; Thomson et al. 2010), and in Nona Bokra to maintaining higher shoot K^+^ content under salt stress (Ren et al. 2005). Bimpong et al. (2014) have reported four grain yield-enhancing QTLs (*qPH8*, *qDTF8*, *qTN8*, and *qTN8*) from Hasawi even for the reproductive stage under saline conditions.

Even though Hasawi is a highly salt-tolerant genotype, it does not have the same tolerance allele as Pokkali and Nona Bokra at *Saltol* and *SKC1* (*OsHKT1;5*), which is a major QTL/gene for salinity tolerance. It has been recently reported that Hasawi is a new source of alleles for salinity tolerance (Bimpong et al. 2014; Bizimana et al. 2017).

The main objectives of our study were to screen IR29/Hasawi-derived RIL populations for seedling-stage salinity tolerance to identify large-effect novel QTLs for traits related to salinity tolerance, to identify epistatic QTLs, to understand the effect of QTL × environment interactions on salinity tolerance, and to identify the RILs with higher salinity tolerance. The novel QTLs identified can be used in marker-assisted QTL pyramiding with other known QTLs to enhance the degree of salinity tolerance in rice.

## Methods

In an attempt to identify robust QTLs for salinity tolerance at the seedling stage using a novel donor, the following activities were carried out.

### Plant materials

A population (about 600) of recombinant inbred lines was developed from a cross between IR29 (salt sensitive) and Hasawi (salt tolerant; IRGC acc. no. 16817) at the International Rice Research Institute (IRRI), Philippines. A set of 300 RILs from this cross was used for phenotyping under controlled environment in the IRRI phytotron in the Philippines (SE Asia – E1). Another set of 300 RILs, different from the one phenotyped in the Philippines, was sent to Africa Rice Center’s Sahel regional station in Senegal (West Africa – E2) and IRRI’s Eastern and Southern Africa office in Tanzania (East Africa – E3) for phenotyping, but under uncontrolled natural environment except for rain protection. Both the E2 and E3 sites received the identical set of RILs.

Each RIL was advanced to constitute the phenotyping population (F_5:6_), in which F_5_ plants were used for genotyping and F_5_-derived F_6_ seedlings/plants were phenotyped at the seedling stage for salinity tolerance at all three phenotyping sites. We followed selective mapping for the study, which is a well-established and robust method for QTL mapping if resources are limited (Lander and Botstein 1989). A subset of 142 common RILs evaluated in both Senegal and Tanzania was genotyped at IRRI, Philippines. The RILs used for genotyping from the set phenotyped at IRRI were completely different (except for three RILs) from those in the set phenotyped in Senegal and Tanzania. This different RIL set used for phenotyping at IRRI consisted of 155 individuals.

#### Environmental classification

This IR29/Hasawi RIL population was evaluated in three different countries using the nutrient solution culture technique following modified Yoshida nutrient solution (Singh and Flowers 2010) in the IRRI phytotron, Philippines (Southeast Asia), and in open screenhouse conditions in Tanzania (East Africa) and the AfricaRice Sahel regional station in Senegal (West Africa).

### Evaluation of F_5:6_ RILs for salt tolerance

Screening of Hasawi, IR29, and the 300 F_5:6_ RILs for salinity tolerance was carried out in a hydroponic system following IRRI standard protocol (Gregorio et al. 1997). Seeds were heat-treated for 5 days in a convection oven set at 48 °C to break seed dormancy, and, after that, the seeds were placed in petri dishes with two layers of paper towels, moistened with distilled water during 48 h for uniform germination. The germinated seeds were sown one seed per hole on a styrofoam sheet with 96 holes, attached to a nylon net bottom, and the sheet was floated on modified Yoshida nutrient solution (Singh et al. 2010). The seedlings were salinized after 5 days using 6 dS m^−1^ salt (NaCl) concentration (equivalent to about 60 mM NaCl). This concentration was increased to 12 dS m^−1^ (~120 mM) after 2 days of 6 dS m^−1^ treatments to reduce the immediate shock. Each genotype was represented by five seedlings per row of styrofoam and replicated thrice in the experiment. The experiment in the Philippines was conducted in a controlled phytotron with 29/21 °C day and night temperature with 70 ± 10% relative humidity. The screenhouse temperature recorded during the experiments in Senegal ranged from 17 to 28 °C in the morning and from 36 to 44 °C in the afternoon. The mean relative humidity varied between 35% and 95%. However, the experiment in Tanzania was conducted in a screenhouse covered by plastic on top only, with a minimum temperature of 24 °C and maximum of 37 °C. The minimum relative humidity was 51% and the maximum 84%, with natural daylight of about 14 h. The pH of the solution was adjusted and maintained at 5.0 to 5.1 every day with acid (1 N HCl) or base (1 N NaOH). The nutrient solution was renewed once every week to limit the effect of algae and to replenish the nutrients. Scoring as per the standard evaluation system (SES) was used and recorded 12 and 25 days after the imposition of salinity stress to finally score the genotype for overall degree of tolerance. All RILs were monitored and scored based on visual symptoms of salt stress injury. The following IRRI modified standard evaluation system (SES) for rice was used (IRRI 2007).

**Table:** 

Score	Symptom/observation	Degree of tolerance
1	Normal growth, only the old leaves show white tips while no symptoms on young leaves	Highly tolerant
3	Near normal growth, but only leaf tips burn, few older leaves become whitish partially and rolled	Tolerant
5	Growth severely retarded; most leaves severely injured, few young leaves elongating	Moderately tolerant
7	Complete cessation of growth; most leaves dried; only a few young leaves still green	Sensitive
9	Almost all plants dead or dying	Highly sensitive

Other phenotypic parameters such as root and shoot length (RL and SL), shoot fresh weight (FWSht), and shoot dry weight (DWSht) were measured after 25 days.

### Genotyping

#### DNA extraction, quantification, and quality control

We followed the approach of selective genotyping (Lander and Botstein 1989) to increase the efficiency of QTL mapping (Lin and Ritland 1996). Seedling SES injury score (final) was used as the parameter to select the genotypes from extremes and also randomly. Out of 300 genotypes, we picked 142 RILs that comprised the 7 most tolerant genotypes (SES score 1–3) and 34 most sensitive ones (SES score 9), and the rest (101) were random genotypes with intermediate to sensitive SES score (5–7). However, in the Philippines, an almost equal number of tolerant, intermediate, and sensitive genotypes was used for genotyping. Genomic DNA was isolated from young leaves using the CTAB (cetyl trimethyl ammonium bromide) mini-preparation method (Murray and Thompson 1980).

#### Scoring of SNPs and analysis of polymorphism

A chip (indica × indica) comprising 384 SNP markers spread throughout 12 chromosomes of the rice genome was used for the parental polymorphic survey between the two parents (Hasawi and IR29). For each OPA (Oligo Pool All, reagent) run, the final DNA concentration was normalized to 50 ng/μL. For the SNP analysis, the (Illumina 2008) GoldenGate assay (Fan et al. 2003) was performed using VeraCode technology on a BeadXpress Reader according to the manufacturer’s protocol. Briefly, about 250 ng of genomic DNA was used to make biotinylated genomic DNA, which then underwent oligonucleotide hybridization to bind the samples to paramagnetic particles, followed by allele-specific extension and ligation, PCR, hybridization to the VeraCode Bead Plate, and scanning on the BeadXpress Reader. The analysis employed the VC0011439-OPA set of 384 SNP markers designed to be informative across *indica* and *aus* germplasm (Thomson et al. 2012) and was run in the Genotyping Services Laboratory at IRRI (Thomson 2014; http://gsl.irri.org). Raw hybridization intensity data processing was performed using the genotyping module in the BeadStudio package (Illumina, San Diego, CA, USA), followed by allele calling using ALCHEMY software (Wright et al. 2010). Graphical genotyping of both IR29 and Hasawi was performed using Flapjack (https://ics.hutton.ac.uk/flapjack/) software developed by the Scottish Crop Research Institute to check polymorphisms. After the polymorphism survey and filtering for low call rates, we found 194 SNPs as polymorphic (which is about 50%) for analysis.

#### SNP linkage map and QTL analysis

Of the 384 SNPs used for the parental polymorphism survey, 194 that were polymorphic were selected for QTL analysis but we had to drop a large number of SNPs for the construction of a linkage map due to segregation distortion (SD).

We used ICIMapping ver. 4.0.1 software (www.isbreeding.net) for the genetic linkage map construction and QTL analysis. Then, a genetic linkage group was constructed based on recombination frequency and SNP ordering was done using the ordering algorithm of RECORD (without imposing marker order) coming from REcombination Counting and ORDering, proposed by van Os et al. (2005a, 2005b). RECORD was developed to produce accurate marker orders in a relatively short time by employing the total number of observable recombination events between adjacent markers as a target function (Wang et al. 2012), although RECORD is not capable of handling populations with high heterozygous loci (Liu et al. 2014). However, it (RECORD) can deal with BC_1_, F_2_, F_3_, and RIL (in fact, any generation obtained by repeated selfing of a hybrid between homozygous parents) mapping populations (van Os et al. 2005a, 2005b). We chose the best ordering algorithm and rippling criteria for fine tuning of the linkage map, which was not used as the input method. COUNT (number of recombination events) algorithm was used for rippling. Rippling was used for fine-tuning of the ordered chromosomes. The Chi-square (χ^2^) test was performed using whole data on a 1:1 basis. The highly distorted markers with <5% probability of either allele for IR29 or Hasawi were discarded; while less or non-distorted markers were included for the linkage map construction and QTL analysis. Segregation distortion markers (SDM) were removed to increase statistical power. The inclusive composite interval mapping (ICIM-ADD) method was used to identify more precise QTLs. For additive mapping, ICIM-ADD retains all advantages of CIM over IM, and avoids the possible increase of sampling variance and the complicated background marker selection process in CIM. Extensive simulations using different genomes and various genetic models indicate that ICIM has increased detection power, reduced false detection rate, and resulted in less biased estimates of QTL effects than CIM in additive (and dominance) mapping. Extensive simulations also show that ICIM is an efficient method for epistasis mapping, and QTL epistatic networks can be identified no matter whether the two QTL have any additive effects (Wang et al. 2014; Xu 2008). The minimal LOD value required to declare a significant QTL was obtained empirically from 1000 permutation tests (Churchill and Doerge 1994). The proportion of the total phenotypic variance explained (PVE) by each QTL was calculated as R^2^ value (R^2^ = PVE). The QTLs were named based on the nomenclature procedure suggested by McCouch et al. (1997) and McCouch and CGSNL (2008).

The digenic (epistatic) interactions between marker loci were determined and single environment (SE) and multi-environment (ME) joint analyses were performed using the multi-environment trials (MET) program in QTL IciMapping ver. 4.0.1 (Wang et al. 2014) to detect QEIs with LOD thresholds of 3.0. We followed the approach of Zhang et al. (2010) in which only reliable QTLs detected by both single and multi-environment analysis were reported.

## Results

The QTLs and QEIs identified by ICIM in the IR29/Hasawi RIL population were based on phenotypic evaluation in three different environments.

### Correlation analysis between traits in the F_6_ RIL population

Significant negative correlations were observed between the SES and all other parameters related to salt tolerance such as SL, RL, FWsht, and DWsht (Table 1), which is expected because a lower SES score indicates higher tolerance, which is based on seedling survival and vigor. In addition, significant positive correlations were observed among traits other than SES score (except between RL and SL in E1), suggesting the importance of these parameters in mechanisms associated with tolerance of salt stress at the seedling stage as indicated by the higher values for these parameters (Table 1).

**Table 1:** Correlation coefficients among different traits in an F_5_ (RIL) population of a cross between IR29 (salt sensitive) and Hasawi (salt tolerant) at seedling stage under three different environments

Environment	Trait	SES	RL	SL	FWsht
Philippines	RL	−0.45^a^			
	SL	−0.18^b^	0.12^ns^		
	FWsht	−0.76^a^	0.35^a^	0.43^a^	
	DWsht	−0.76^a^	0.34^a^	0.41^a^	0.98^a^
Tanzania	RL	−0.75^a^			
	SL	−0.64^a^	0.70^a^		
	FWsht	−0.79^a^	0.67^a^	0.64^a^	
	DWsht	−0.86^a^	0.75^a^	0.74^a^	0.84^a^
Senegal	RL	−0.44^a^			
	SL	−0.46^a^	0.33^a^		
	FWsht	−0.50^a^	0.40^a^	0.46^a^	
	DWsht	−0.34^a^	0.19^b^	0.33^a^	0.45^a^

*SES* Standard evaluation system score based on salt stress symptoms, *SL* Shoot length, *RL* Root length, *FWsht* Shoot fresh weight, *DWsht* Shoot dry weight, *ns* non-significant

^a^ and ^b^ indicate significance at the 1% and 5% level, respectively

### Genotypic analysis

We used single nucleotide polymorphic markers to determine the polymorphism between the two parents (Hasawi and IR29). In all, 194 SNPs out of 384 (50.52%) showed polymorphism between the parents. After removing distorted markers, 145 SNP markers were used in E1 and 135 markers in E2 and E3 to perform genetic linkage analysis. They were distributed throughout the rice genome and covered a total length of 1655 cM using E1 genotyping data and 1662 cM using E2 and E3 genotyping data, with an average interval of 11.4 and 12.3 cM between markers, respectively. The highest marker density was found on chromosome 1 (23), with an average interval of 10 cM (Additional file 1: Table S1).

### Marker segregation distortion analysis

The expected genotypic ratio of 1:1 in the F5:6 RIL population for homozygous IR29:homozygous Hasawi allele varied with three categories. First, with no segregation distortion that accounted for 40 SNPs (in E1) and 37 SNPs (E2 and E3); second, very highly distorted markers (<5% probability of either allele) varied from Mendelian segregation ratio for this RIL population, and less distorted markers (105 for E1 and 98 for E2 and E3). Only non-distorted and less distorted markers were used for mapping studies. When selective mapping is followed, some of the segregation distortions happen due to a sampling effect as well. The whole population was subjected to χ^2^ significance (*P* = 0.05) before analyzing the data. This was done to avoid false linkages from the expected Mendelian segregation ratio. Indeed, little SD for the specific markers is expected to have the effect of the allele through marker-trait association; otherwise, this association will not be reflected as a significant QTL. Hackett and Broadfoot (2003) suggested that segregation distortion causes very little effect on both marker order and map length.

### QTL analysis

**Fig. 1:**
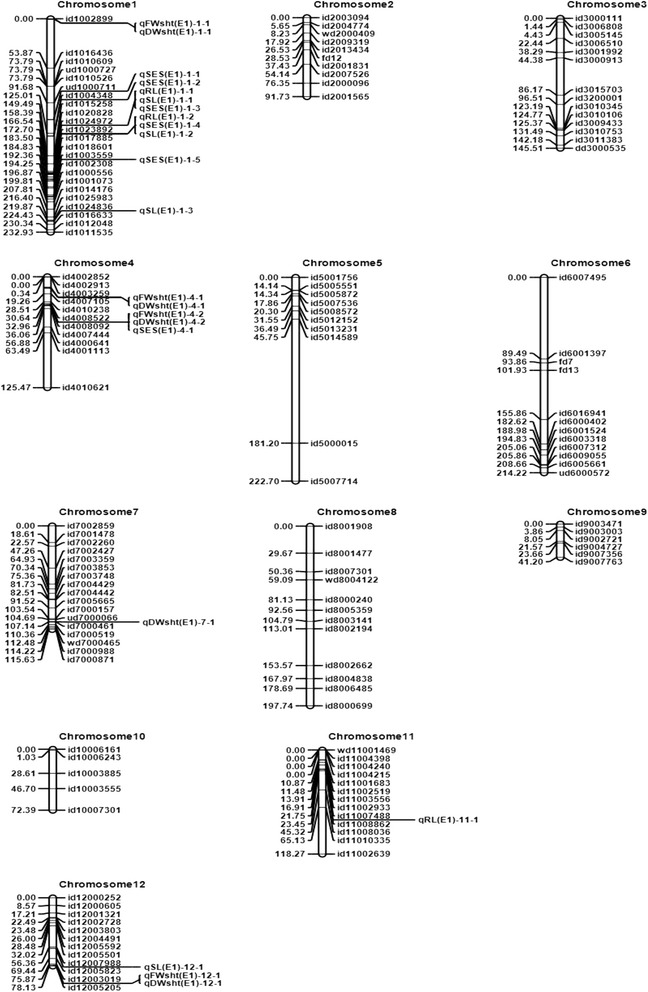
Genetic linkage maps of the 12 chromosomes constructed using ordering algorithm (RECORD) based on an IR29/Hasawi RIL population phenotyped in the phytotron at IRRI (E1). The names of the SNP markers with position are listed to the right and the approximate locations of the QTLs detected for salinity tolerance are shown to the left of the chromosomes

**Fig. 2:**
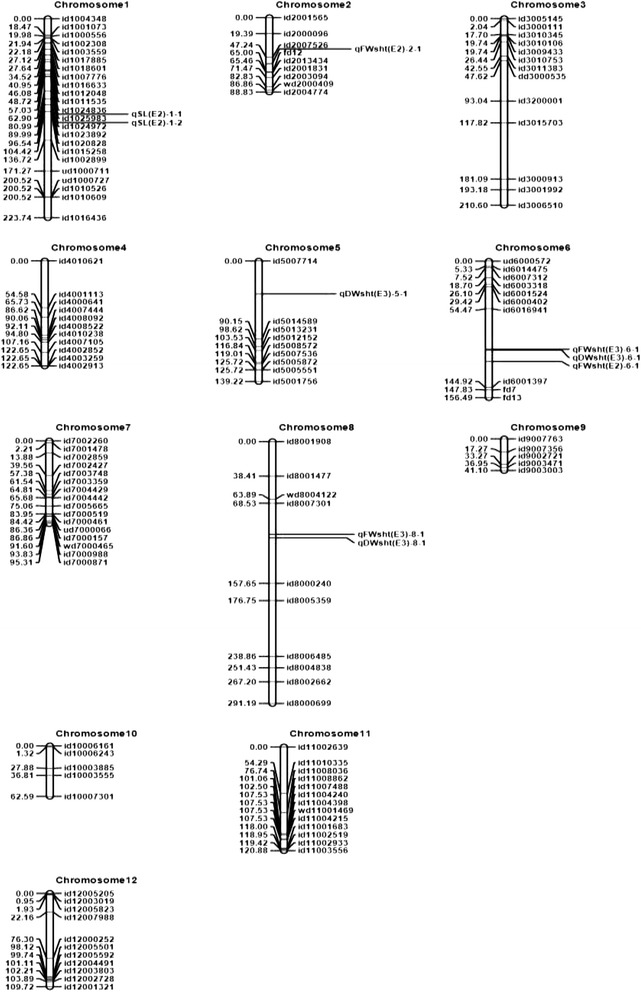
Genetic linkage maps of the 12 chromosomes constructed using ordering algorithm (RECORD) based on an IR29/Hasawi RIL population phenotyped in Senegal (E2) and Tanzania (E3). The names of the SNP markers with position are listed to the right and the approximate locations of the QTLs detected for salinity tolerance are shown to the left of the chromosomes

Inclusive composite interval mapping (ICIM-ADD) was employed to identify putative QTLs. However, the QTLs reported here were identified after constructing the genetic linkage map using ordering algorithm (RECORD) instead of the input method. A total of 34 different QTLs were identified in three diverse saline environments (Table 2). The QTLs conferring tolerance of salinity at the seedling stage were identified on 10 chromosomes: chromosome 1 (*qSES1.1, qSES1.2, qSES1.3, qSES1.4, qSES1.5, qSL1.1, qSL1.2, qSL1.3, qSL1.4, qRL1.1, qRL1.2, qFWsht1.1, qFWsht1.2, qFWsht1.3, qDWsht1.1, qDWsht1.2, qDWsht1.3*); chromosome 2 (*qFWsht2.1*); chromosome 3 (*qRL3.1*); chromosome 4 (*qSES4.1, qFWsht4.1, qFWsht4.2, qDWsht4.1, qDWsht4.2*); chromosome 5 (*qDWsht5.1*); chromosome 6 (*qFWsht6.1, qDWsht6.1*); chromosome 7 (*qDWsht7.1*); chromosome 8 (*qFWsht8.1, qDWsht8.1*); chromosome 11 (*qRL11.1*); and chromosome 12 (*qSL12.1, qFWsh12.1*, *qDWsht12.1*). One QTL (*qFWsht 6.1*) was identified in two different environments (E2 and E3) within the same chromosomal location. The details of the QTLs are presented in Table 2. The QTLs with a large effect are also illustrated on the molecular linkage map (Fig. 1 based on E1 and Fig. 2 based on E2 and E3).

**Table 2:** QTLs for traits related to salt tolerance during seedling stage in an IR29/Hasawi RIL population in three environments

Trait	QTL	Chr	Position (cM)	Marker interval	Allele	LOD	PVE (%)	Add	E	Method
SES	*qSES1.1*	1	110	ud1000711-id1004348	Hasawi	3.2	10.7	−0.31	E1	ICIM
	*qSES1.2*	1	128	id1004348-id1015258	Hasawi	4.9	8.8	−0.27	E1	ICIM
	*qSES1.3*	1	170	id1024972-id1023892	Hasawi	17.5	39.9	−0.58	E1	IM, ICIM
	*qSES1.4*	1	175	id1023892-id1017885	Hasawi	20.6	42.3	−0.60	E1	IM, −ICIM
	*qSES1.5*	1	194	id1003559-id1002308	IR29	3.8	5.4	0.24	E1	-IM, ICIM
	*qSES4.1*	4	32	id4008522-id4008092	Hasawi	3.8	5.8	−0.23	E1	IM, ICIM
SL	*qSL1.1*	1	59	id1024836-id1025983	Hasawi	4.4	18.5	−2.13	E2	IM, ICIM
	*qSL1.2*	1	169	id1024972-id1023892	Hasawi	6.5	17.9	−3.25	E1	IM, ICIM
	*qSL1.3*	1	177	id1023892-id1017885	Hasawi	5.7	19.5	−3.40	E1	IM, ICIM
	*qSL1.4*	1	221	id1024836-id1016633	IR29	5.6	15.3	3.36	E1	IM, ICIM
	*qSL12.1*	12	58	id12007988-id12005823	IR29	3.3	7.2	2.23	E1	IM, ICIM
RL	*qRL1.1*	1	168	id1024972- id1023892	Hasawi	4.8	14.3	−1.09	E1	IM, ICIM
	*qRL1.2*	1	175	id1023892-id1017885	Hasawi	4.2	13.5	−1.06	E1	IM, ICIM
	*qRL3.1*	3	104	id3200001-id3010345	Hasawi	3.2	21.9	−1.53	E1	IM
	*qRL11.1*	11	22	id11007488-id11008862	IR29	5.0	14.7	2.11	E1	IM, ICIM
FWsht	*qFWsht1.1*	1	0	id1002899-id1016436	Hasawi	5.5	10.2	−0.09	E1	IM, ICIM
	*qFWsht1.2*	1	175	id1023892-id1017885	Hasawi	3.6	21.1	−0.12	E1	IM
	*qFWsht1.3*	1	194	id1003559-id1002308	Hasawi	4.5	13.8	−0.11	E1	IM
	*qFWsht2.1*	2	55	id2007526-fd12	Hasawi	3.1	27.2	−0.12	E2	IM, ICIM
	*qFWsht4.1*	4	12	id4003259-id4007105	IR29	6.1	28.9	0.14	E1	IM, ICIM
	*qFWsht4.2*	4	32	id4008522-id4008092	IR29	4.3	8.8	0.08	E1	IM, ICIM
	*qFWsht6.1*	6	115	id6016941-id6001397	IR29	3.2	37.8	0.22	E2	IM, ICIM
	*qFWsht6.1*	6	115	id6016941-id6001397	IR29	3.3	47.1	0.56	E3	IM, ICIM
	*qFWsht8.1*	8	105	id8007301-id8000240	IR29	3.5	47.1	0.55	E3	IM, ICIM
	*qFWsht12.1*	12	78	id12003019-id12005205	IR29	4.4	8.0	0.09	E1	ICIM
DWsht	*qDWsht1.1*	1	0	id1002899-id1016436	Hasawi	3.4	5.5	−0.02	E1	IM, ICIM
	*qDWsht1.2*	1	175	id1023892-id1017885	Hasawi	3.4	19.6	−0.03	E1	IM
	*qDWsht1.3*	1	194	id1003559-id1002308	Hasawi	3.9	12.0	−0.02	E1	IM
	*qDWsht4.1*	4	13	id4003259-id4007105	IR29	8.3	30.1	0.03	E1	IM, ICIM
	*qDWsht4.2*	4	32	id4008522-id4008092	IR29	3.2	5.9	0.02	E1	IM, ICIM
	*qDWsht5.1*	5	38	id5007714-id5014589	IR29	3.2	46.9	0.12	E3	IM, ICIM
	*qDWsht6.1*	6	115	id6016941-id6001397	IR29	3.7	48.4	0.12	E3	IM, ICIM
	*qDWsht7.1*	7	107	ud7000066-id7000461	IR29	3.3	5.7	0.02	E1	IM, ICIM
	*qDWsht8.1*	8	105	id8007301-id8000240	IR29	3.8	47.2	0.12	E3	IM, ICIM
	*qDWsht12.1*	12	78	id12003019-id12005205	IR29	4.6	7.5	0.02	E1	IM, ICIM

*Chr* Chromosome, *LOD* Log of odds, *Add* Additive effect, *IM* Interval mapping, *ICIM* Inclusive composite interval mapping, *E* Environment, *E1* Philippines, *E2* Senegal, *E3* Tanzania, *MI*: Marker interval, *PVE* Phenotypic variation of the rice RIL population explained by each QTL

### QTLs for salinity tolerance at seedling stage

#### Overall phenotypic performance (SES)

Six QTLs (*qSES1.1*, *qSES1.2*, *qSES1.3, qSES1.4, qSES1.5,* and *qSES4.1*) were evaluated by the SES in controlled (evaluated for salinity tolerance under phytotron conditions) Environment-1 (Philippines) with significant LOD value (3.2–20.6) and PVE ranging from 5.4% to 42.3%. The Hasawi allele increased the overall phenotypic performance and reduced SES visual scores at five loci except *qSES1.5,* where the allelic effect comes from IR29 (Table 2). The QTLs (*qSES1.3* and *qSES1.4*) on the long arm of chromosome 1 are located in a similar region as *qSL1.2* and *qSL1.3* in E1. *qSES1.4*, located between markers id1023892 and id1017885 on chromosome 1, had the largest PVE (42.3%) and was additive in nature.

#### Shoot length

Four QTL regions (*qSL1.1, qSL1.2, qSL1.3, qSL1.4*) with significant LOD value (3.3–6.5) were identified for SL on the long arm of chromosome 1 and one QTL (*qSL12.1*) located at 58 cM on chromosome 12 in E1 had PVE ranging from 7.2% to 19.5%. *qSL1.2* is located between SNP markers id1024972 and id1023892 and co-located with two other QTLs (*qSES1.3* and *qRL1.1*). *qSL1.3* is detected between id1023892 and id1017885 and shared a common genomic region with four other large-effect QTLs (*qSES1.4, qRL1.2, qFWsht1.2*, and *qDWsht1.2*) on the long arm of chromosome 1 (Table 2).

#### Root length

Four root-length QTLs (*qRL1.1, qRL1.2, qRL3.1*, and *qRL11.1*) with significant LOD value (3.2–5.0) were detected on chromosomes 1, 3, and 11 in the Philippines. These QTLs had PVE of 14.3%, 13.5%, 21.9%, and 14.7%, respectively. Hasawi alleles contributed to longer root length. Two QTLs (*qRL1.1* and *qRL1.2*) located between id1024972-id1023892 and id1023892-id1017885 were additive**.**

#### Shoot fresh weight

Nine genomic regions were identified for shoot fresh weight with significant LOD of 3.1 to 6.1. Three FWsht QTLs (*qFWsht1.1, qFWsht1.2, qFWsht1.3*) were located on chromosome 1 with LOD value of 3.6–5.5 and they accounted for PVE of 10.2%, 21.1%, and 13.8%, respectively, and Hasawi had positive effects on shoot fresh weight in the Philippines. This QTL (*qFWsht1.2*) was co-located with four other important genomic regions (*qSES1.4, qRL1.2, qSL1.3*, and *qDWsht1.2*) on the long arm of chromosome 1. One QTL (*qFWsht6.1*) was identified in both E2 and E3 with positive effects from IR29. *qFWsht12.1* shared a common location with *qDWsht12.1* at 78 cM on chromosome 12 (Table 2).

#### Shoot dry weight

In the Philippines, ICIM-ADD detected 10 significant QTLs (*qDWsht1.1, qDWsht1.2, qDWsht1.3, qDWsht4.1, qDWsht4.2, qDWsht5.1, qDWsht6.1, qDWsht7.1, qDWsht8.1,* and *qDWsht12.1*) with LOD value ranging from 3.2 to 8.3 for shoot dry weight on seven chromosomes (1, 4, 5, 6, 7, 8, and 12) near markers id1002899, id1023892, id1003559, id4003259, id4008522, id5007714, id6016941, ud7000066, id8007301, and id12003019, which explained PVE ranging from 5.5% to 48.4%. Out of seven QTLs, three located on chromosome 1 (*qDWsht1.1* at zero (0) cM*; qDWsht1.2* at 175 cM*; qDWsht1.3* at 194 cM), two on chromosome 4 (*qDWsht4.1* at 12 cM and *qDWsht4.2* at 32 cM), one on chromosome 8 (*qDWsht8.1* at 105 cM), and one on chromosome 12 (*qDWsht12.1* at 78 cM) were observed to share common genomic regions with QTLs conferring for shoot fresh weight and SES score. *qDWsht6.1* (E3) is co-localized with QTLs *qFWsht6.1* (E2) and *qFWsht6.1* (E3) and has PVE of 48.4% (Table 2).

As per the multi-locus analysis, QTLs identified on different chromosomes are considered independent of each other, and their effects were generally additive in nature. For example, the major QTLs for SES score on chromosome 1 (flanked by markers id1024972-id1023892, with PVE of 39.9%, and another located between id1023892 and id1017885, with PVE of 42.3%) and chromosome 4 (flanked by markers id4008522-id4008092, with PVE of 5.8%) together accounted for 88.0% of the total PVE observed in the study from the Philippines. Likewise, the combined effect of three QTLs (*qDWsht1.2*, R^2^ = 19.6%; *qDWsht1.3*, R^2^ = 12.0%; *qDWsht4.1*, R^2^ = 30.1%) explained 61.7% of the total phenotypic variance for overall shoot dry weight, which is a part of the phenotypic performance that affects SES score in the Philippines. SES score is based on plant vigor and higher plant vigor comes from vigorous shoot growth; hence, a lower SES score and more shoot biomass will eventually give more shoot dry weight and this clearly indicates that these are traits indirectly linked to each other.

#### QTL interactions for seedling-stage salinity tolerance

##### (i) Epistatic interactions

**Fig. 3:**
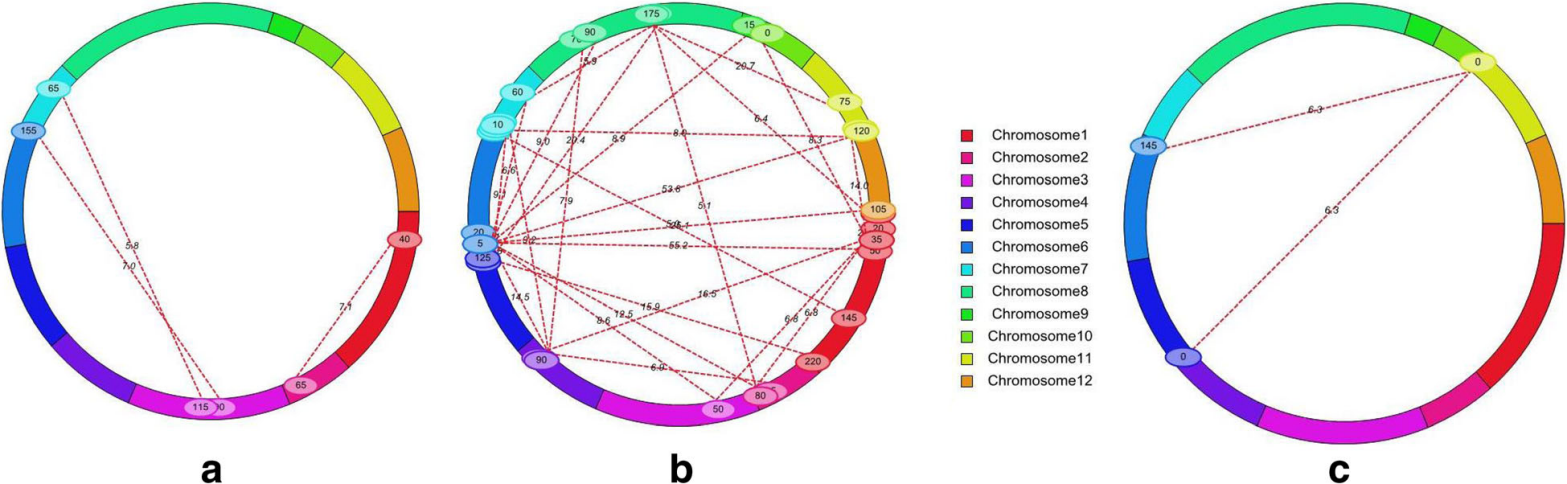
Cyclic illustrations of epistatic QTLs for salt tolerance indices: (**a**) shoot fresh weight, (**b**) shoot dry weight, and (**c**) SES score with QTL × environment interactions on linkage groups by ICIM. The dotted lines indicate the interacting marker pairs located on the same or different chromosomes with corresponding LOD score due to epistatic effect. Chromosomes 1 (40 cM) and 2 (*qFWsht2.1,* 65 cM) showed interaction between QTL and background loci for shoot fresh weight (**b**), whereas chromosomes 1 (35 cM) and 4 (95 cM) showed interaction between complementary loci for DWsht (see Table 3)

A two-way test to detect epistatic interactions between marker loci was performed for single environment (SE) and multi-environment (ME) analyses with stringent threshold LOD of 5.0 using the ICIM-EPI method of ICIM version 4.0.1 software for all traits. The SE analysis detected 49 significant digenic interactions (2 in E1, 31 in E2, and 16 in E3) located across 12 different chromosomes for SES score, SL, FWsht, and DWsht, whose PVE ranged from 13.6% to 44.9% in E1, from 16.0% to 75.9% in E2, and from 21.9% to 74.9% in E3 (Additional file 1: Table S2). Out of 49 digenic interactions, we identified 13 interactions between QTLs and background loci, 36 interactions between complementary loci, and no interaction between QTLs observed (Additional file 1: Table S2). The ME analysis identified a total of 30 significant interactions consisting of one interaction for SL, two marker loci intervals (MI) for SES score, three MI for FWsht, and 24 intervals for DWsht that were spread across 10 different chromosomes (1, 2, 3, 4, 5, 6, 7, 8, 11, and 12) (Table 3). Two types of digenic interactions were identified (Table 3; Fig. 3): (I) interaction between the QTL (marker interval fd12-id2013434; *qFWsht2.1*) on chromosome 2 for FWsht and background loci (such as marker interval id1007776-id1016633) on chromosome 1 with LOD of 7.1 for shoot fresh weight (Fig. 3a); and (II) interaction between complementary locus 1 (35 cM; MI: id1007776-id1016633) on chromosome 1 and 95 cM; MI: id4010238-id4007105 on chromosome 4 for shoot dry weight (Fig. 3b) and interaction between background loci at MI: id6001397-fd7 (145 cM) on chromosome 6 and MI: id11002639- id11010335) on chromosome 11 (0 cM) with LOD of 6.3 and PVE of 7.5% for SES score (Table 3; Fig. 3c). Interaction between QTLs is not found in this study. Four marker pairs had a significant effect on the final phenotype through the interaction between the QTL and background loci and 26 significant interactions between complementary loci, thus indicating strong interaction effects. The interaction component study on shoot dry weight revealed that one of the marker intervals (id1004348-id1001073) on chromosome 1 hosted a main-effect QTL (*qDWsht1.1*) that interacted with background loci on chromosome 8 at marker interval id8000240-id8005359 to express the phenotype with 4.0% of explainable variation due to total interaction components. Out of 26 type II interactions (the interaction between complementary loci), the marker intervals (ud6000572-id6014475) on chromosome 6 interacted with other background loci on chromosome 11 at marker interval id11002933-id11003556 to express the phenotype with a very high LOD for both epistatic interaction (LOD: 53.7; the highest PVE of 14.1%) and QTL × E interaction (LOD = 31.0, PVE of 8.1%), suggesting the higher probability of occurrence for digenic and QTL × E interactions (Table 3). More epistatic interactions seem to be present for shoot dry weight than for others as indicated by the multiple dotted lines connecting the chromosomes.

**Table 3:** Epistasis for traits related to salinity tolerance in an IR29/Hasawi RIL population in two different environments (E2 and E3)

Chr 1	Position 1 (cM)	Marker interval at position 1	Chr 2	Position 2 (cM)	Marker interval at position 2	LOD			PVE (%)			AA × E2	AA × E3	TI
						Epi	AA	QEI	Epi	AA	QEI			
SL: Shoot length														
2	20	id2000096-id2007526	8	35	id8002194-id8001477	7.0	2.4	3.5	9.3	4.6	4.7	0.87	−0.87	C
SES														
5	0	id5007714-id5014589	11	0	id11002639-id11010335	6.3	4.3	2.0	7.8	7.2	0.6	−0.72	0.72	C
6	145	id6001397-fd7	11	0	id11002639-id11010335	6.3	4.1	2.1	7.5	7.1	0.4	−0.72	0.72	C
FWsht: Shoot fresh weight														
1	40	id1007776-id1016633	2	65	fd12-id2013434	7.1	4.1	3.0	5.4	3.3	2.1	0.05	−0.05	B
3	90	dd3000535-id3200001	6	155	fd7-fd13	7.0	3.2	3.7	8.6	3.8	4.8	−0.16	0.16	C
3	115	id3200001-id3015703	7	65	id7004429-id7004442	5.8	3.1	2.6	7.8	4.0	3.7	0.12	−0.12	C
DWsht: Shoot dry weight														
1	35	id1007776-id1016633	2	80	id2001831-id2003094	6.8	1.0	5.8	3.0	0.4	2.6	−0.04	0.04	C
2	65	fd12-id2013434	4	90	id4007444-id4008092	6.9	3.7	3.2	2.1	2.1	0.0	−0.04	0.04	B
1	35	id1007776-id1016633	4	95	id4010238-id4007105	16.5	4.8	11.7	7.1	2.5	4.7	−0.07	0.07	C
1	220	id1010609-id1016436	5	120	id5007536-id5005872	15.9	3.8	12.0	1.2	1.5	0.0	−0.07	0.07	B
1	50	id1011535-id1024836	6	5	ud6000572-id6014475	55.2	29.1	26.1	5.6	5.6	0.0	−0.14	0.14	C
2	80	id2001831-id2003094	6	5	ud6000572-id6014475	12.5	2.3	10.2	8.0	1.1	6.8	−0.06	0.06	C
4	90	id4007444-id4008092	6	5	ud6000572-id6014475	14.5	3.3	11.1	8.1	1.6	6.5	−0.06	0.06	C
5	120	id5007536-id5005872	6	5	ud6000572-id6014475	68.8	39.1	29.7	7.9	7.9	0.1	−0.16	0.16	C
6	5	ud6000572-id6014475	6	20	id6003318-id6001524	19.1	5.2	13.8	4.8	3.9	0.9	−0.06	0.06	C
1	145	id1002899-ud1000711	7	0	id7002260-id7001478	5.0	1.6	3.3	1.0	0.8	0.2	0.03	−0.03	C
5	125	id5007536-id5005872	7	5	id7001478-id7002859	9.1	0.0	9.1	2.6	2.7	0.0	0.06	−0.06	C
4	90	id4007444-id4008092	7	15	id7002859-id7002427	9.2	2.9	6.2	1.0	1.0	0.0	0.05	−0.05	C
6	5	ud6000572-id6014475	7	60	id7003748-id7003359	6.6	3.8	2.7	5.1	1.6	3.5	−0.05	0.05	C
4	90	id4007444-id4008092	8	70	id8007301-id8000240	7.9	2.0	5.9	1.2	1.0	0.2	0.04	−0.04	C
6	5	ud6000572-id6014475	8	90	id8007301-id8000240	9.0	0.0	9.5	2.5	1.0	1.5	0.05	−0.05	C
1	0	id1004348-id1001073	8	175	id8000240-id8005359	6.3	4.3	2.0	4.0	4.0	0.1	0.02	−0.02	B
2	80	id2001831-id2003094	8	175	id8000240-id8005359	5.0	0.4	4.6	3.5	0.2	3.3	0.03	−0.03	C
7	60	id7003748-id7003359	8	175	id8000240-id8005359	5.9	1.7	4.1	3.9	1.1	2.8	0.03	−0.03	C
5	125	id5007536-id5005872	8	180	id8005359-id8006485	20.3	4.9	15.4	5.8	3.5	2.3	0.06	−0.06	C
8	175	id8000240-id8005359	11	75	id11010335-id11008036	20.7	4.9	15.7	2.8	2.8	0.0	0.08	−0.08	C
1	35	id1007776-id1016633	11	115	id11004215-id11001683	14.0	2.6	11.4	4.2	1.3	2.9	−0.06	0.06	C
6	5	ud6000572-id6014475	11	120	id11002933-id11003556	53.7	22.7	31.0	14.1	5.9	8.1	−0.12	0.12	C
7	10	id7001478-id7002859	11	120	id11002933-id11003556	8.0	0.0	8.6	5.0	1.1	3.9	−0.05	0.05	C
6	5	ud6000572-id6014475	12	105	id12002728-id12001321	25.0	7.2	17.8	3.7	2.8	0.9	−0.09	0.09	C

AA by E represents the estimated additive effect of epistatic QTLs and QTL **×** environment interactions (QEI) under two environments, respectively; its positive value indicates that IR29 has the positive allele and is just the opposite for negative values; its positive value indicates that two-loci genotypes are the same as those in the parent IR29 having positive effects, whereas the two-loci recombinants have negative effects (from Hasawi); PVE (epistatic QTLs), PVE (AA), and PVE (QEI) represent the PVE by the epistatic QTLs, additive/main-effect QTLs, and QTL **×** environment interactions, respectively. *Epi* Epistatic, *TI*: Type of interaction, *A* interaction between QTLs, *B* interaction between QTLs and background loci, *C* interaction between complementary loci

##### (ii) QTL × environment interactions (QEI)

Out of 30 digenic interactions (1 for SL, 2 for SES score, 3 for FWsht, and 24 for DWsht) identified by the combined analysis of the multi-environment phenotypic values under two environmental conditions, 18 significant QEI were identified with LOD of 5.8 to 31.0 and PVE of 0.2% to 8.1% for DWsht (Table 3; Fig. 3). Three QTLs (*qSES4.1, qSL1.1,* and *qDWsht8.1*) were identified through combined analysis of both E2 and E3 (Additional file 1: Table S3). Importantly, *qSES4.1, qSL1.1,* and *qDWsht8.1* corresponded to main-effect QTLs such as *qSES4.1, qSL1.1,* and *qDWsht8.1,* respectively (Table 2).

#### Identification of tolerant RILs and use in breeding

There were significant differences in SL, RL, FWsht, DWsht, and SES score between the two parents under salinity (Additional file 1: Table S4). Hasawi had higher SL, RL, FWsht, and DWsht than the sensitive IR29. Transgressive segregation was observed for these traits and the heritabilities of SL, RL, FWsht, DWsht, and SES score ranging from 50% to 74% are high (Additional file 1: Table S4), indicating the repeatability/precision of the trials. Thus, four RILs (IR91477–13–1-1-1, IR91477–64–1-1, IR91477–76–1-1, and IR91477–250–1-1) with high salt tolerance and that had multiple introgression of the related QTLs were selected for evaluation and could be used as potential donors (pre-breeding materials) for further breeding programs (Table 4).

**Table 4:** Phenotypic values of traits for salt tolerance among selected RILs and parents common in Senegal and Tanzania with their related QTLs

Selected RILs	SL (cm)	RL (cm)	FWsht (g)	DWsht (g)	SES score	QTLs
IR91477–13–1-1-1	42.5	16.7	0.98	0.24	**3.0**	*qSES1.4, qSES4.1, qSL1.3*
IR91477–16–1-1-1	43.5	20.9	1.07	0.25	4.3	*qSES1.4, qSL1.3, qFWsht6.1*
IR91477–64–1-1	35.8	20.8	1.18	0.26	4.0	*qSES1.4, qSL1.3, qRL1.1, qFWsht6.1*
IR91477–76–1-1	36.4	22.2	0.79	0.20	4.3	*qSES1.4, qSES4.1, qDWsht6.1, qFWsht4.2*
IR91477–250–1-1	37.3	17.5	1.02	0.27	3.5	*qSES1.4, qSL1.3, qFWsht4.2, qFWsht6.1*
IR29 (sensitive check)	20.5	11.3	0.61	0.13	8.0	
Hasawi (tolerant check)	40.0	22.9	1.30	0.28	3.0	
Heritability (%)	72.5	78.5	55.0	59.3	68.6	

## Discussion

Most studies on seedling-stage salinity tolerance have been carried out using common donors such as Pokkali and Nona Bokra. There have been fewer attempts to identify new donors for salinity tolerance, to identify novel QTLs, and to understand epistatic interaction effects among the QTLs and other loci, and also the effect of environmental interaction on QTL expression for salinity tolerance. In this study, we used Hasawi, a landrace from Saudi Arabia found to have strong adaptability to salinity and drought stress environments (Al-Mssallem et al. 2011; Bizimana et al. 2017). We made crosses using Hasawi, a tolerant donor, and IR29, the recipient parent. IR29 is an internationally recognized salinity-sensitive rice check variety. We developed F_5:6_ RILs and screened them under three diverse environments across Asia and Africa.

The major findings of this study were five QTL hot-spot (co-localized) regions identified on five different chromosomes for multiple QTLs such as chromosome 1 (*qSES1.3, qSES1.4, qSL1.2, qSL1.3, qRL1.1, qRL1.2, qFWsht1.2, qDWsht1.2*), chromosome 4 (*qSES4.1, qFWsht4.1, qFWsht4.2, qDWsht4.1, qDWsht4.2*), chromosome 6 (*qFWsht6.1, qDWsht6.1*), chromosome 8 (*qFWsht8.1, qDWsht8.1*), and chromosome 12 (*qSL12.1, qFWsht12.1, qDWsht12.1*); five salinity-tolerant RILs identified with multiple QTL introgression and that could be used directly in breeding programs; novel additive robust QTLs on the long arm of chromosome 1 for SES score, SL, RL, FWsht, and DWsht in a similar region; and one QTL (*qFWsht6.1*) detected in E2 and E3 for FWsht. Thirty-four QTLs identified through ICIM-ADD and IM-ADD mapping are additive because of the use of a permanent mapping population RIL with no or few heterozygotes; consequently, a dominant effect was absent.

SES score is a visual parameter for assessing the tolerance of seedlings under salinity stress. The lower the SES score (1 or 3), the higher the tolerance, whereas a higher SES score (7 or 9) suggests sensitivity. An SES score of 5 indicates moderate tolerance. Significant negative correlations were observed for SES score with all the other four parameters (SL, RL, FWsht, and DWsht) (Table 1). This is obvious because seedlings can be scored with low SES only if they have attained higher shoot length and high vigor, which means long root length, although higher SES scores could be given to plants with poor vigor and growth. This suggests that all four growth-related parameters directly relate to visually based SES scores. The negative associations observed between SES score and plant growth attributes clearly demonstrate the significance and the detrimental effects of high Na^+^ accumulation in plant tissue under saline conditions. The most common salt injury symptoms in rice are leaf tip burning, early senescence, and complete necrosis, particularly among sensitive varieties such as IR29. The detrimental effects of salt stress on the growth and yield of rice genotypes are well documented in several earlier reports (Flowers and Yeo 1981, 1995; Gregorio and Senadhira 1993; Ashraf et al. 1999; Ismail et al. 2007; Munns and Tester 2008; Ding et al. 2010; Singh et al. 2010; Bimpong et al. 2016). The mostly positive significant correlations among SL, RL, FWsht, and DWsht suggest that these traits ultimately contribute to seedling-stage salinity tolerance.

This finding is in agreement with earlier findings that wide genetic variation exists for the traits RL and DWsht in rice under saline conditions at the seedling stage (Maiti et al. 2006; Al-Amin et al. 2013; Bimpong et al. 2014). It has also been reported that salinity stress leads to negative root growth and development (Roy et al. 2002; Rodrigues et al. 2002).

### Stable genomic regions for salinity tolerance in multi-environments

Genetic linkage maps, based on pair-wise distance estimates, have emerged as pivotal tools for locating genes or QTLs. The analysis of recombination events from marker segregation data is especially helpful when a large number of markers segregate in a single mapping population. But, mapping larger numbers of markers also exponentially increases the potential orders of these loci on a chromosome. Hence, advanced and efficient algorithms are required to achieve near-perfect ordering of large numbers of loci. REcombination Counting and ORDering (RECORD) (van Os et al. 2005a, 2005b) is a faster, more accurate method for ordering of loci on genetic linkage maps and it performs especially well in regions of maps with high marker density. Therefore, RECORD was used to identify the best marker order in each linkage group and to generate a linkage map of rice. A total of 34 QTL regions were identified for five traits in our study across three environments. Eight QTLs for SES score, SL, RL, FWsht, and DWsht (*qSES1.3, qSES1.4, qSL1.2, qSL1.3, qRL1.1, qRL1.2, qFWsht1.2,* and *qDWsht1.2*) were identified within 170–175 cM (Table 2) on the long arm of chromosome 1. Although they are not at an identical position, it looks like an important region. *qSES1.4,* which is an additive QTL and has a high LOD value with PVE of 42.3%, is also identified in the same region. The identified QTLs (*qSES1.3, qSES1.4, qSL1.2, qSL1.3, qRL1.1, qRL1.2, qFWsht1.2,* and *qDWsht1.2*) on chromosome 1 in our study are located in different regions (170–175 cM) than the earlier reported QTLs on chromosome 1 such as *Saltol* (10.6–11.5 Mb) and *SKC1* (11.46 Mb) (Bonilla et al. 2002; Ren et al. 2005; Kim et al. 2009). It is interesting to note that most of the QTLs controlling seedling-stage salinity tolerance are reported on chromosome 1 in different studies and in the current study also we identified QTLs for all the studied traits except DWsht. A large number of QTLs for salinity tolerance have already been reported on the short arm of chromosome 1 (Claes et al. 1990; Flowers et al. 2000; Takehisa et al. 2004; Lin et al. 2004; Ren et al. 2005; Zhang et al. 2008; Sabouri et al. 2009; Ammar et al. 2009; Thomson et al. 2010; Bimpong et al. 2014).

The novel QTLs identified on chromosome 1 (*qSES1.3, qSES1.4, qSL1.2, qSL1.3, qRL1.1, qRL1.2*) from Hasawi needs further investigation to identify the candidate genes and to understand the molecular mechanisms conferring early seedling-stage salinity tolerance in rice, which could be through similar or different physiological pathways.

The other common QTL detected in two environments is *qFWsht6.1* (E2 and E3), which is co-localized with *qDWsht6.1* (E3) (Table 2). *qDWsht8.1* for DWsht shared a common genomic region (105 cM) with *qFWsht8.1* on chromosome 8. Indeed, both the traits are very closely associated. Three QTLs (*qSES4.1, qFWsht4.2,* and *qDWsht4.2*) are located in a similar position (32 cM) on chromosome 4 as the cluster at the marker interval of id4008522-id4008092. They may be working as QTL clusters of large QTLs due to co-localization. This region needs to be saturated with more markers for further fine mapping.

It was observed in multi-locus analysis that, if the pooled effects of some major QTLs such as those for SES score on different chromosomes (the pooled PVE of three SES score QTLs is 88.0%, with *qSES1.3,* R^2^ = 39.9%*; qSES1.4*, R^2^ = 42.3%; *qSES4.1*, R^2^ = 5.8%) and for DWsht (the pooled PVE of three DWsht QTLs is 61.7%, with *qDWsht1.2,* R^2^ = 19.6%; *qDWsht1.3,* R^2^ = 12.0%; *qDWsht4.1*, R^2^ = 30.1%) are considered as for one trait, then the overall phenotypic manifestation of their individual effects seems to be not as strong as expected (Table 2). This observation might be due to (i) co-localization of the QTLs, (ii) less-than-additive epistatic interactions among QTLs as suggested by Eshed and Zamir (1996), and (iii) some of the QTLs may be affecting the common traits through similar developmental processes.

Importantly, out of a total of 34 QTLs, 9 main-effect QTLs identified for five different traits such as 2 QTLs for SES score (*qSES1.3, qSES1.4*)*,* 2 for shoot length (*qSL1.2, qSL1.3*), 2 for root length (*qRL1.1, qRL1.2*), 1 for shoot fresh weight (*qFWsht6.1*), and 2 for shoot dry weight (*qDWsht1.2, qDWsht6.1*) could be focused on for further details and fine mapping for the important traits linked with salt tolerance.

### Digenic interaction

Epistasis is a major factor underlying quantitative traits (Caicedo et al. 2004). In our study, potential epistatic interactions between marker loci identified on 10 different chromosomes revealed 49 significant digenic interactions (2 interactions for E1, 31 for E2, and 16 for E3) through single environment analysis for SES score, shoot length, root length, shoot fresh weight, and shoot dry weight, with quite a wide range of PVE. But, we are not elaborating on this as our focus is on QTL interactions with environments; hence, we worked with multi-environment analysis. The ME analysis illustrated 17 marker intervals resulting in 30 two-way interactions (Table 3; Fig. 3); these interactions significantly influenced the traits, suggesting that epistasis is an important component of the genetic basis for complex traits, including tolerance of salt stress. Individually, all the complementary/background markers reported in Table 3 had no significant effect on the trait alone (otherwise, they would have mapped as reliable large-effect QTLs), but resulted in an enhanced effect when combined and interacting with QTLs and other markers. However, only one significant digenic interaction each was identified for SL, two interactions for SES score, and three interactions for FWsht, as several large-effect QTLs were detected for these traits. Some researchers have suggested the presence of significant epistatic interactions among QTL-linked or -unlinked markers (Cocherham and Zeng 1996; Eshed and Zamir 1996; Li et al. 1997; Hossain et al. 2015). So, there is a need to assess the importance of epistatic gene interactions as this complicates the genotype-phenotype relationships; in addition, different computing models used in analyzing epistasis vary and give different results (Malmberg et al. 2005). The interaction effect may enhance or reduce the expected manifestation of overall QTL effect depending upon the degree and direction of the interaction. Type II interactions (between complementary loci) had relatively higher PVE than type I interactions, probably because the trait manifests itself only through interaction between two complementary loci as no main-effect QTL is involved. The influence of epistatic QTL interactions alone explained the trait variation, ranging from 2.1% to 14.1%, which could be crucial when the threshold limits for salinity tolerance of a variety are to be enhanced to withstand environmental stress.

Studies in *Arabidopsis* and rice (Malmberg et al. 2005; Mei et al. 2005; Wang et al. 2011) have suggested that epistatic QTL effects are more important than additive QTL effects for fitness traits, for example, the loss of effect of the *Saltol* region in the introgression process to develop near-isogenic lines (NILs) (Thomson et al. 2010). The clean NILs were susceptible to salinity stress compared with NILs having interaction between *Saltol* and other background loci from the donor parent Pokkali. By contrast, studies designed to explicitly model epistatic interactions in other crops such as maize revealed that epistasis is of little or only moderate importance for quantitative traits (Schon et al. 2004; Mihaljevic et al. 2005; Blanc et al. 2006). These contrasting results might be due in part to the relative importance of epistatic effects in predominantly inbreeding or predominantly outcrossing species, and in part to differences in modeling procedures.

Several chromosomal regions were associated with more than one trait, indicating linkage or pleiotropic effects. For instance, three QTLs (*qSES1.3, qSL1.2,* and *qRL1.1*) located at 170 cM linked with MI: id1024972-id1023892 and five other QTLs such as *qSES1.4, qSL1.3, qRL1.2, qFWsht1.2,* and *qDWsht1.2* are closely associated with MI: id1023892-id1017885 at 175 cM. One SNP (id1023892) is found to be common in both marker intervals, which looks like a major common SNP in the region located on the long arm of chromosome 1, conferring salt tolerance. It is important to note that the same QTL might contribute to several traits associated with a specific phenotype because of closely associated traits. Hence, epistatic effects and pleiotropy can play a notable role in the interaction and function of a QTL; the presence of a QTL with a very small effect may have a large effect on a regulatory pathway (Koyama et al. 2001). Further characterization of this region by fine mapping and the identification of the genes underlying its tolerance will shed more light on whether the same set of genes or an entirely different set of linked genes governs these phenotypes.

### QTL × environment interaction (QEI) for salinity tolerance

QTL × environment interaction plays an important role in adaptation to changing environments. QEI is particularly high in self-pollinated plants such as rice (Jain and Marshall 1967; Wang et al. 2014) and the complex epistatic interactions and QTL **×** environment interaction effects are important in controlling salt tolerance (Wang et al. 2011). In comparison to main-effect QTLs whose LOD threshold was kept at 3.0, the threshold was kept at >5.0 as the threshold for two-way interactions to identify only very significant QTLs. All the interactions were significant when LOD (epistasis) and LOD (QEI) ranged from 5.8 to 68.8 and 5.8 to 31.0, respectively. However, the LOD (AA) of 12 interactions is non-significant (LOD = 0.0 to 2.9) (Table 3). This suggests that these 12 interactions have less additive effect than QTL **×** environment interaction effects to express the phenotype. Combined LOD partitioned into PVE for additive-effect QTL and QTL × E interactions suggests potential enhancement of stress tolerance by a genotype for a specific environment through the sum of overall manifestation effects of QEI. Thus, QEI has a huge influence on salinity tolerance as the degree of salinity is dependent on environmental factors (temperature, humidity, rainfall), crop season, and crop growth stage. Besides this, the negative AA × E2 value (Table 3) indicates that interaction with Hasawi alleles rather than IR29 alleles may be one of the reasons for making the seedlings more tolerant of salinity stress. The relatively lower contribution of QTL × E interaction through additive components does not eliminate the possibility and importance of dominance or epistatic QTLs or interactions between the QTLs and the environment. E1 was the controlled environment whereas E2 and E3 were uncontrolled natural environments except for rain protection, and this was expected to have more QTL × E interaction, but the experiment infers that there is not a high order of interaction component but enough for affecting and elevating the threshold tolerance limits. Nine additive major QTLs that were identified (seven additive QTLs in the Philippines, one in both Tanzania and Senegal, and one in Senegal) and 30 epistatic QTLs that were identified by joint analyses suggest that epistatic QTLs and QTL × environment interactions are important components for FWsht, DWsht, SES score, and SL. Our investigation revealed a significant combined effect of epistatic interaction and QTL × E interaction with high PVE, but, on further dissection, we found more epistatic interaction than QTL × E interaction probably because of the higher heritable trait (Additional file 1: Table S2; Tables 3 and 4). Thus, major QTL effects, QEI, and epistatic interactions need to be considered together to improve selection efficiency using genomic-assisted breeding (Zhao et al. 2010; Liu et al. 2014).

### Comparison of the new QTL loci with previously mapped QTLs

Results of comparative analysis of the QTL positions identified in the study compared with the QTL positions identified in earlier studies as being associated with salinity tolerance at various growth stages are shown. Rice cultivars grown in saline environments are most sensitive at both the vegetative and reproductive stages. However, the relationship between tolerances at the two stages is poor, suggesting that they are regulated by different processes and genes (Singh and Flowers 2010; Hossain et al. 2015; Rahman et al. 2016; Ahmadizadeh et al. 2016). The major QTL *Saltol,* derived from salt-tolerant landrace Pokkali, has been mapped on chromosome 1. This QTL confers salt tolerance at the vegetative stage and explains between 39.2% and 43.9% of the PVE in the original RIL population (Bonilla et al. 2002), but further studies found that *Saltol* alone does not work as a robust QTL (Thomson et al. 2010). A gene for salt tolerance at the vegetative stage, *SKC1*, has been identified in the same region from Nona Bokra and positionally cloned (Ren et al. 2005). *SKC1* maintains K^+^ homoeostasis in the salt-tolerant cultivar under salt stress, and the gene encodes a member of HKT-type transporters. This gene turns out to be a protein in the HKT family that exclusively mediates K^+^ and Na^+^ translocation between roots and shoots, thereby regulating K^+^/Na^+^ homeostasis in the shoots, resulting in improved salt tolerance (Ren et al. 2005). The eight novel QTLs (*qSES1.3, qSES1.4, qSL1.2, qSL1.3, qRL1.1, qRL1.2, qFWsht1.2,* and *qDWsht1.2*) responsible for seedling-stage salinity tolerance on the long arm of chromosome 1 as reported in our study were found to be very different from *SKC1* and *Saltol*. These eight novel QTLs span a region of 170 to 175 cM. There is a need to further test the stability of the identified QTLs being expressed before drawing a conclusion.

Koyama et al. (2001) identified 10 QTLs for five shoot traits related to salt tolerance: Na^+^ concentration (one QTL) at 74 cM on chromosome 1; K^+^ concentration (three QTLs) on chromosomes 4, 6, and 9; Na^+^ concentration (two QTLs) on chromosomes 4 and 6; K^+^ concentration (two QTLs) on chromosomes 1 (at 56 cM) and 4; and Na^+^-K^+^ ratio in shoots (two QTLs) on chromosomes 1 (at 74 cM) and 4. In our study, eight QTLs were identified for SES score and shoot and root traits and are located on chromosome 1 but in a different position (~175.0 cM) on the long arm versus the short arm of the chromosomes in the previous studies. Lin et al. (2004) detected five QTLs for four traits associated with salt tolerance in roots and three QTLs for three shoot traits associated with salt tolerance, but none of these QTLs have the same map locations as any of the QTLs identified here across environments, suggesting that most of these QTLs are novel and could be important for breeding. Wang et al. (2012) reported five additive QTLs, for Na^+^ in shoots (*qSNC9*), K^+^ in shoots (*qSKC1* and *qSKC9*), K^+^ in roots (*qRKC4*), and for salt tolerance rating (*qSTR7*), as new salt tolerance loci. However, *qSES4.1, qFWsht4.1, qFWsht4.2, qDWsht1.1, qDWsht1.2,* and *qDWsht7.1* in E1 were identified that shared similar chromosomal positions in our study.

The QTLs that co-localize in a similar region (such as id1023892 marker) probably indicate some functional relatedness among them. This major QTL cluster might also have pleiotropic effects on other traits. The cluster of QTLs on chromosome 1 for different traits, such as SES score, SL, and RL (Fig. 1), was also supported by the strong correlations observed among these traits (Table 1). This clustering of loci and correlation of effects can be attributed to different linked QTLs occurring on the same segment or pleiotropic effects of a single QTL. High-resolution mapping is required to determine whether pleiotropic effects are present.

The QTLs identified in this study that overlap with others mapped previously fall into two categories: (i) QTLs that share a similar map position and are mapped to the same trait, and (ii) QTLs that share a similar map position but are mapped to a different trait. However, we found a tight cluster of QTLs localizing around 170–175.0 cM on chromosome 1, and three QTLs (*qSES4.1, qFWsht4.2,* and *qDWsht4.2*) on chromosome 4 at 32.0 cM and two QTLs (*qFWsht6.1, qDWsht6.1*) on chromosome 6 at 115.0 cM, which may be considered as novel loci. Several studies have indicated that many genomic loci controlling important rice traits are clustered in the same chromosome regions (Cai and Morishima 2002; Angaji 2008; Hossain et al. 2015). These major loci should be targeted for pyramiding through MABC (Singh et al. 2007; Thomson et al. 2010). Many QTLs for SES score, shoot length, root length, shoot fresh weight, and shoot dry weight were identified. The ones accounting for higher LOD and PVE could subsequently be used for QTL pyramiding after the development of low-cost diagnostic molecular markers linked to them.

### Responses of the RILs and their parental lines to salt stress

The most crucial step of QTL mapping for salt tolerance in rice is the evaluation of salt tolerance (Wang et al. 2011). We selected five salt-tolerant RILs based on visual phenotypic score (SES score) that are commonly tolerant in two environments (Senegal and Tanzania). Salinity has large effects on crop growth, yield, and productivity (Tester and Davenport 2003; Munns and Tester 2008; Munns and Gilliham 2015). Initial vigor that directly relates to higher shoot and root length and fresh and dry weight of shoots through faster growth at early seedling stage could reduce the Na^+^ concentration in plant tissues probably because of a dilution effect besides other salt-tolerance mechanisms operating in plants.

## Conclusions

Salinity tolerance is a complex quantitative trait and previous studies established its strong association with visual symptoms (as indicated by the SES score) and other traits such as shoot length, root length, shoot fresh weight, and shoot dry weight. Overall phenotypic performance reflected by SES scores is determined by these key traits. We identified genomic regions on chromosomes 1, 2, 3, 4, 5, 6, 7, 8, 11, and 12 that are associated with salinity tolerance at the seedling stage by affecting SES scores, shoot length, root length, shoot fresh weight, and shoot dry weight. Thirty-four QTLs for the five traits were detected on 10 chromosomes. The QTLs (*qSES1.3, qSES1.4, qSL1.2, qSL1.3, qRL1, qRL1.2, qFWsht1.2,* and *qDWsht1.2*) detected on chromosome 1 could be of much interest and termed as novel QTLs as, unlike earlier reported *Saltol* and *SKC1*, they are on the long arm of chromosome 1. The study also detected 30 digenic two-way interactions through ME analysis that are quite important for gene expression, especially for complex traits such as salinity tolerance. Significant QTL × environment interaction for FWsht, DWsht, SES score, and SL indicated as high as a 8.1% contribution for phenotypic manifestation through interaction between QTLs and background loci, or complementary loci. The robust QTLs, digenic interactions, and QEI could be good targets for more detailed QTL studies, fine mapping, and subsequent pyramiding to develop highly tolerant varieties that could lead to the development of improved rice varieties for salt-affected areas where salt stress is a major impediment to rice production.

## Additional files

Additional file 1: **Table S1.** Linkage map information. **Table S2.** Digenic interactions/epistatic QTLs (LOD >5.0) using ICIM-EPI method in single environments E1, E2, and E3. **Table S3.** Significant QTLs detected in two environments (E2 and E3) for traits related to salt tolerance in an IR29/Hasawi RIL population by inclusive composite interval mapping (ICIM) through combined analysis. **Table S4.** Responses of different salt-tolerant RIL genotypes of rice and their parents under salt stress at seedling stage in (a) the Philippines, (b) Senegal, and (c) Tanzania. (DOCX 47 kb)

## Abbreviations

DWsht: Dry weight of shoot
FWsht: Fresh weight of shoot
PVE: Phenotypic variance explained
QTLs: Quantitative trait loci
RL: Root length
SDM: Segregation distortion markers
SES score: Standard evaluation system (SES) score
SL: Shoot length
