# Supplementary material for: Mapping QTLs using a novel source of salinity tolerance from Hasawi and their interaction with environments in rice
Source: Rice (N Y). 2017 Nov 2;10:47. doi: 10.1186/s12284-017-0186-x (PMC5668218; doi:10.1186/s12284-017-0186-x)
Supplement: Supplementary file 1 — Table S1. Linkage map information. Table S2. Digenic interactions/epistatic QTLs (LOD >5.0) using ICIM-EPI method in single environments E1, E2, and E3. Table S3. Significant QTLs detected in two environments (E2 and E3) for traits related to salt tolerance in an IR29/Hasawi RIL population by inclusive composite interval mapping (ICIM) through combined analysis. Table S4. Responses of different salt-tolerant RIL genotypes of rice and their parents under salt stress at seedling stage in (a) the Philippines, (b) Senegal, and (c) Tanzania. (DOCX 47 kb) [file 12284_2017_186_MOESM1_ESM.docx]

# Supplementary Table 1: Linkage map information.

**(a) Map information for E1:**

| Linkage map information: | | |
| --- | --- | --- |
| Chromosome ID | Number of markers | Length (cM) |
| 1 | 23 | 232.93 |
| 2 | 10 | 91.73 |
| 3 | 14 | 145.51 |
| 4 | 11 | 125.47 |
| 5 | 10 | 222.70 |
| 6 | 12 | 214.22 |
| 7 | 17 | 115.63 |
| 8 | 12 | 197.74 |
| 9 | 6 | 41.20 |
| 10 | 5 | 72.39 |
| 11 | 13 | 118.27 |
| 12 | 12 | 78.13 |
| Whole genome | 145 | 1655.92 |

**(b) Map information for E2 and E3:**

|  | | |
| --- | --- | --- |
| Chromosome ID | Number of markers | Length (cM) |
| 1 | 23 | 223.74 |
| 2 | 9 | 88.83 |
| 3 | 13 | 210.60 |
| 4 | 11 | 122.65 |
| 5 | 9 | 139.22 |
| 6 | 10 | 156.49 |
| 7 | 16 | 95.31 |
| 8 | 10 | 291.19 |
| 9 | 5 | 41.10 |
| 10 | 5 | 62.59 |
| 11 | 13 | 120.88 |
| 12 | 11 | 109.72 |
| Whole genome | 135 | 1662.32 |

**Supplementary Table 3:** Digenic interactions/epistatic QTLs (LOD >5.0) using ICIM-EPI method in single environments E1, E2, and E3.

| Traits | Chr 1 | Position 1 | Marker interval at position 1 | Chr 2 | Position 2 | Marker interval at position 2 | LOD | PVE (%) | Add1 | Add2 | Dom1 | Dom2 | TI |
| --- | --- | --- | --- | --- | --- | --- | --- | --- | --- | --- | --- | --- | --- |
| **E1: Philippines** | | |  |  |  |  |  |  |  |  |  |  |  |
| SES score | **1** | **170** | **id1024972-id1023892** | **5** | **190** | **id5000015-id5007714** | **5.1** | **44.9** | **-0.98** | **-3.69** | **0.82** | **-1.16** | **B** |
|  | **6** | **130** | **id2004774-fd12** | **7** | **105** | **ud7000066-id7000461** | **6.1** | **13.6** | **0.58** | **-0.19** | **1.07** | **0.06** | **B** |

**E2: Senegal**

| Trait | Chr 1 | Position 1 | Marker interval at position 1 | Chr 2 | Position 2 | Marker interval at position 2 | LOD | PVE (%) | Add1 | Add2 | Add by Add | TI |
| --- | --- | --- | --- | --- | --- | --- | --- | --- | --- | --- | --- | --- |
| SES score | 5 | 0 | id5007714-id5014589 | 11 | 0 | id11002639-id11010335 | 5.3 | 16.0 | -1.60 | -1.33 | -1.69 | C |
|  | 6 | 145 | id6001397-fd7 | 11 | 0 | id11002639-id11010335 | 5.5 | 16.6 | -1.60 | -1.32 | -1.71 | C |
| FWsht | 4 | 90 | id4007444-id4008092 | 8 | 50 | id8001477-wd8004122 | 5.2 | 30.8 | -0.14 | 0.13 | -0.20 | C |
| DWsht | 1 | 60 | id1024836-id1025983 | 2 | 70 | id2013434-id2001831 | 42.1 | 69.8 | -0.27 | -0.26 | 0.26 | C |
|  | 1 | 20 | id1000556-id1002308 | 4 | 90 | id4007444-id4008092 | 41.5 | 69.3 | -0.26 | -0.26 | 0.26 | C |
|  | 3 | 110 | id3200001-id3015703 | 4 | 90 | id4007444-id4008092 | 41.9 | 73.6 | 0.24 | -0.28 | -0.28 | C |
|  | 2 | 70 | id2013434-id2001831 | 5 | 125 | id5007536-id5005872 | 42.8 | 69.8 | -0.25 | -0.28 | 0.27 | C |
|  | 3 | 135 | id3015703-id3000913 | 6 | 20 | id6003318-id6001524 | 43.8 | 74.0 | 0.24 | -0.28 | -0.28 | C |
|  | **5** | **35** | **id5007714-id5014589** | **6** | **20** | **id6003318-id6001524** | **41.7** | **72.9** | **0.27** | **-0.25** | **-0.24** | **B** |
|  | **1** | **30** | **id1018601-id1007776** | **6** | **115** | **id6016941-id6001397** | **43.9** | **72.3** | **-0.26** | **0.26** | **-0.28** | **B** |
|  | **6** | **5** | **ud6000572-id6014475** | **6** | **110** | **id6016941-id6001397** | **30.0** | **52.6** | **-0.28** | **0.31** | **-0.27** | **B** |
|  | **2** | **80** | **id2001831-id2003094** | **6** | **115** | **id6016941-id6001397** | **43.5** | **72.6** | **-0.27** | **0.25** | **-0.28** | **B** |
|  | 1 | 145 | id1002899-ud1000711 | 7 | 5 | id7001478-id7002859 | 42.7 | 70.2 | 0.27 | 0.25 | 0.27 | C |
|  | 4 | 90 | id4007444-id4008092 | 7 | 5 | id7001478-id7002859 | 43.2 | 70.9 | -0.26 | 0.25 | -0.27 | C |
|  | 5 | 125 | id5007536-id5005872 | 7 | 5 | id7001478-id7002859 | 45.4 | 71.0 | -0.27 | 0.25 | -0.27 | C |
|  | 6 | 95 | id6016941-id6001397 | 7 | 5 | id7001478-id7002859 | 44.0 | 71.2 | 0.26 | 0.26 | 0.28 | C |
|  | 2 | 15 | id2001565-id2000096 | 8 | 190 | id8005359-id8006485 | 45.4 | 75.4 | -0.29 | 0.23 | -0.29 | C |
|  | 3 | 25 | id3009433-id3010753 | 8 | 190 | id8005359-id8006485 | 46.6 | 75.9 | -0.29 | 0.23 | -0.29 | C |
|  | 4 | 90 | id4007444-id4008092 | 8 | 190 | id8005359-id8006485 | 47.2 | 75.6 | -0.28 | 0.23 | -0.29 | C |
|  | 5 | 125 | id5007536-id5005872 | 8 | 190 | id8005359-id8006485 | 46.8 | 74.6 | -0.28 | 0.24 | -0.28 | C |
|  | 7 | 10 | id7001478-id7002859 | 9 | 35 | id9002721-id9003471 | 35.9 | 69.4 | 0.26 | 0.26 | 0.26 | C |
|  | **6** | **115** | **id6016941-id6001397** | **9** | **40** | **id9003471-id9003003** | **40.6** | **72.7** | **0.24** | **0.27** | **0.28** | **B** |
|  | 8 | 195 | id8005359-id8006485 | 9 | 40 | id9003471-id9003003 | 41.5 | 72.4 | 0.24 | 0.27 | 0.27 | C |
|  | 5 | 120 | id5007536-id5005872 | 10 | 20 | id10006243-id10003885 | 31.0 | 69.3 | -0.26 | -0.26 | 0.26 | C |
|  | **6** | **110** | **id6016941-id6001397** | **10** | **25** | **id10006243-id10003885** | **37.9** | **69.4** | **0.26** | **-0.26** | **-0.26** | **B** |
|  | 1 | 100 | id1020828-id1015258 | 11 | 120 | id11002933-id11003556 | 34.3 | 69.3 | -0.26 | -0.26 | 0.26 | C |
|  | **6** | **110** | **id6016941-id6001397** | **11** | **120** | **id11002933-id11003556** | **43.6** | **73.7** | **0.24** | **-0.28** | **-0.28** | **B** |
|  | 7 | 5 | id7001478-id7002859 | 11 | 120 | id11002933-id11003556 | 42.9 | 71.4 | 0.25 | -0.27 | -0.27 | C |
|  | 8 | 195 | id8005359-id8006485 | 11 | 120 | id11002933-id11003556 | 45.5 | 74.6 | 0.24 | -0.28 | -0.28 | C |
|  | **6** | **110** | **id6016941-id6001397** | **12** | **85** | **id12000252-id12005501** | **41.0** | **74.9** | **0.23** | **0.29** | **0.29** | **B** |
|  | 8 | 190 | id8005359-id8006485 | 12 | 85 | id12000252-id12005501 | 44.3 | 74.4 | 0.24 | 0.28 | 0.28 | C |

**E3: Tanzania**

| Trait | Chr 1 | Position 1 | Marker interval at position 1 | Chr 2 | Position 2 | Marker interval at position 2 | LOD | PVE (%) | Add1 | Add2 | Add by Add | TI |
| --- | --- | --- | --- | --- | --- | --- | --- | --- | --- | --- | --- | --- |
| SL | 2 | 15 | id2001565-id2000096 | 8 | 35 | id8001908-id8001477 | 5.1 | 34.1 | 1.87 | -0.58 | 5.73 | C |
| FWsht | 1 | 40 | id1007776-id1016633 | 2 | 65 | fd12-id2013434 | 5.4 | 22.1 | -0.37 | -0.31 | 0.46 | C |
|  | 1 | 190 | ud1000711-ud1000727 | 3 | 40 | id3010753-id3011383 | 5.9 | 48.2 | 0.31 | 0.17 | 0.37 | C |
|  | 3 | 25 | id3009433-id3010753 | 6 | 155 | fd7-fd13 | 5.3 | 27.0 | 0.44 | 0.28 | 0.43 | C |
|  | 4 | 55 | id4001113-id4000641 | 8 | 80 | id8007301-id8000240 | 5.0 | 39.0 | -0.23 | 0.29 | -0.36 | C |
|  | 3 | 70 | dd3000535-id3200001 | 11 | 85 | id11008036-id11008862 | 5.7 | 49.7 | 0.19 | 0.35 | 0.43 | C |
| DWsht | 1 | 40 | id1007776-id1016633 | 2 | 65 | fd12-id2013434 | 5.4 | 21.9 | -0.08 | -0.07 | 0.09 | C |
|  | 1 | 5 | id1004348-id1001073 | 3 | 70 | dd3000535-id3200001 | 6.3 | 50.0 | -0.07 | 0.051 | -0.10 | C |
|  | 3 | 40 | id3010753-id3011383 | 5 | 25 | id5007714-id5014589 | 6.3 | 49.7 | 0.05 | 0.076 | 0.09 | C |
|  | **2** | **0** | **id2001565-id2000096** | **5** | **40** | **id5007714-id5014589** | **5.4** | **48.8** | **0.05** | **0.12** | **0.05** | **B** |
|  | **1** | **150** | **id1002899-ud1000711** | **6** | **115** | **id6016941-id6001397** | **5.0** | **40.6** | **0.09** | **0.07** | **0.11** | **B** |
|  | 3 | 70 | dd3000535-id3200001 | 6 | 135 | id6016941-id6001397 | 6.3 | 49.9 | 0.06 | 0.08 | 0.12 | C |
|  | 5 | 50 | id5007714-id5014589 | 11 | 25 | id11002639-id11010335 | 5.1 | 69.1 | 0.09 | 0.13 | 0.03 | C |
|  | **8** | **105** | **id8007301-id8000240** | **11** | **35** | **id11002639-id11010335** | **5.3** | **74.9** | **0.11** | **0.10** | **0.02** | **B** |
|  | 3 | 70 | dd3000535-id3200001 | 11 | 85 | id11008036-id11008862 | 7.0 | 54.1 | 0.04 | 0.08 | 0.10 | C |
|  | 5 | 45 | id5007714-id5014589 | 12 | 100 | id12005592-id12004491 | 5.0 | 46.6 | 0.05 | 0.06 | 0.08 | C |

TI: Type of interaction, A: interaction between QTLs, B: interaction between QTLs and background loci, C: interaction between complementary loci

**Supplementary Table 4:** Significant QTLs detected in two environments (E2 and E3) for traits related to salt tolerance in an IR29/Hasawi RIL population by inclusive composite interval mapping (ICIM) through combined analysis.

| Traits | Chr. | Position  (cM) | Marker interval | LOD | LOD (A) | LOD  (A by E) | PVE | PVE (A) | PVE  (A by E) | Add | A by E2 | A by E3 |
| --- | --- | --- | --- | --- | --- | --- | --- | --- | --- | --- | --- | --- |
| *qSL1.1* | 1 | 59.0 | id1024836-id1025983 | 4.2 | 1.2 | 3.0 | 2.5 | 1.7 | 0.8 | -1.02 | -0.68 | 0.68 |
| *qSES4.1* | 4 | 65.0 | id4004493-id4006135 | 4.0 | 2.5 | 1.4 | 6.0 | 3.9 | 2.1 | 0.16 | -1.66 | -0.65 |
| *qDWsht8.1* | 8 | 110 | id8007301-id8000240 | 7.7 | 1.8 | 5.9 | 15.7 | 4.7 | 10.9 | -0.26 | -0.24 | 0.98 |

**Supplementary Table 5:** Responses of different salt-tolerant RIL genotypes of rice and their parents under salt stress at seedling stage in (a) the Philippines, (b) Senegal, and (c) Tanzania.

(a) Philippines

| Genotypes | Shoot length (cm) | Root length (cm) | Shoot fresh weight (g) | Shoot dry weight (g) | SES score |
| --- | --- | --- | --- | --- | --- |
| IR91477-61-1-1-1-1 | 43.5 | 16.7 | 0.98 | 0.24 | 4.6 |
| IR91477-65-1-1-1-1 | 43.5 | 20.9 | 1.07 | 0.25 | 4.3 |
| IR91477-84-1-1-1-1 | 36.9 | 17.1 | 0.99 | 0.27 | 3.6 |
| IR91477-105-1-1-1-1 | 47.4 | 17.4 | 1.23 | 0.26 | 4.3 |
| IR91477-137-1-1-1-1 | 39.7 | 17.0 | 1.04 | 0.24 | 4.3 |
| IR91477-148-1-1-1-1 | 35.8 | 20.8 | 1.18 | 0.26 | 4.0 |
| IR91477-167-1-1-1-1 | 41.6 | 16.9 | 0.94 | 0.25 | 5.0 |
| IR91477-249-1-1 | 41.0 | 15.6 | 1.08 | 0.28 | 3.6 |
| IR91477-261-1-1-1-1 | 40.7 | 15.7 | 0.95 | 0.26 | 4.0 |
| IR91477-266-1-1-1-1 | 36.4 | 22.2 | 0.79 | 0.19 | 4.3 |
| IR91477-313-1-1-1-1 | 34.3 | 14.1 | 0.96 | 0.22 | 3.3 |
| IR91477-355-1-1-1-1 | 40.3 | 22.8 | 1.32 | 0.33 | 3.0 |
| IR91477-358-1-1-1-1 | 35.4 | 14.1 | 1.15 | 0,27 | 3.3 |
| IR91477-420-1-1-1-1 | 30.9 | 20.8 | 1.21 | 0.24 | 3.0 |
| IR91477-421-1-1-1-1 | 35.4 | 16.6 | 0.79 | 0.20 | 4.3 |
| IR91477-429-1-1-1-1 | 35.5 | 22.5 | 1.24 | 0.29 | 4.0 |
| IR91477-493-1-1-1-1 | 37.1 | 17.2 | 0.88 | 0.22 | 4.0 |
| IR91477-518-1-1-1-1 | 32.5 | 11.9 | 0.81 | 0.20 | 4.3 |
| IR91477-607-1-1-1-1 | 37.8 | 21.5 | 1.17 | 0.28 | 3.6 |
| IR91477-685-1-1-1-1 | 37.3 | 17.4 | 1.02 | 0.27 | 4.0 |
| IR29 (sensitive check) | 19.0 | 11.3 | 0,61 | 0.13 | 7.6 |
| Hasawi (tolerant check) | 38.4 | 22.2 | 1.20 | 0.28 | 3.0 |
| Significance |  |  |  |  |  |
| Genotype | *** | *** | ** | ** | *** |
| LSD (*P* <0.05) | 0.93 | 1.70 | 0.26 | 0.04 | 0.88 |
| CV (%) | 3.5 | 8.0 | 15.1 | 10.1 | 13.4 |
| Heritability (%) | 70.1 | 63.1 | 73.2 | 55.1 | 62.0 |

CV, coefficient of variation

(b) Senegal

| Genotypes | Shoot length (cm) | Root length (cm) | Shoot fresh weight (g) | Shoot dry weight (g) | SES score |
| --- | --- | --- | --- | --- | --- |
| IR91477-13-1-1-1 | 41.53 | 16.73 | 0.98 | 0.24 | 4.67 |
| IR91477-16-1-1-1 | 43.50 | 19.97 | 1.07 | 0.25 | 4.33 |
| IR91477-25-1-1 | 35.97 | 17.10 | 0.99 | 0.27 | 3.67 |
| IR91477-37-1-1 | 45.40 | 17.43 | 1.23 | 0.26 | 4.33 |
| IR91477-61-1-1 | 38.73 | 16.07 | 1.04 | 0.25 | 4.33 |
| IR91477-64-1-1 | 35.87 | 19.00 | 1.18 | 0.26 | 4.00 |
| IR91477-69-1-1 | 41.60 | 16.97 | 0.94 | 0.26 | 5.00 |
| IR91477-71-1-1 | 41.00 | 15.60 | 1.08 | 0.29 | 3.67 |
| IR91477-72-1-1 | 40.70 | 16.70 | 0.96 | 0.26 | 4.00 |
| IR91477-76-1-1 | 36.43 | 22.23 | 0.79 | 0.20 | 4.33 |
| IR91477-84-1-1 | 34.30 | 14.70 | 0.96 | 0.22 | 3.33 |
| IR91477-106-1-1 | 40.37 | 22.87 | 1.32 | 0.33 | 3.00 |
| IR91477-115-1-1 | 35.40 | 14.17 | 1.15 | 0.27 | 3.33 |
| IR91477-121-1-1 | 30.93 | 20.80 | 1.21 | 0.24 | 3.00 |
| IR91477-138-1-1 | 35.47 | 16.70 | 0.79 | 0.20 | 4.33 |
| IR91477-181-1-1 | 35.53 | 20.53 | 1.24 | 0.30 | 4.00 |
| IR91477-183-1-1 | 37.10 | 17.27 | 0.89 | 0.22 | 4.00 |
| IR91477-190-1-1 | 32.57 | 11.90 | 0.82 | 0.20 | 4.33 |
| IR91477-220-1-1 | 37.83 | 21.53 | 1.17 | 0.29 | 3.67 |
| IR91477-250-1-1 | 34.37 | 15.47 | 1.02 | 0.27 | 3.50 |
| IR29 (sensitive check) | 20.50 | 11.30 | 0.61 | 0.13 | 8.0 |
| Hasawi (tolerant check) | 40.00 | 22.90 | 1.30 | 0.28 | 3.0 |
| Significance |  |  |  |  |  |
| Genotype | *** | *** | *** | *** | *** |
| LSD (*P* <0.05) | 3.20 | 2.80 | 0.26 | 0.11 | 1.30 |
| CV (%) | 7.5 | 7.2 | 33.1 | 30.1 | 23.4 |
| Heritability (%) | 70.0 | 67.2 | 50.2 | 54.5 | 55.2 |

CV, coefficient of variation

(c) Tanzania

| Genotypes | Shoot length (cm) | Root length (cm) | Shoot fresh weight (g) | Shoot dry weight (g) | SES score |
| --- | --- | --- | --- | --- | --- |
| IR91477-13-1-1 | 44.23 | 24.33 | 2.03 | 0.58 | 1.33 |
| IR91477-16-1-1 | 33.93 | 18.43 | 1.38 | 0.36 | 3.33 |
| IR91477-19-1-1 | 53.31 | 27.91 | 1.60 | 0.28 | 2.67 |
| IR91477-36-1-1 | 24.78 | 15.64 | 1.66 | 0.34 | 2.67 |
| IR91477-44-1-1 | 33.92 | 21.07 | 2.54 | 0.55 | 1.00 |
| IR91477-61-1-1 | 46.26 | 21.18 | 1.45 | 0.26 | 3.33 |
| IR91477-64-1-1 | 44.93 | 16.56 | 0.99 | 0.31 | 4.33 |
| IR91477-76-1-1 | 38.89 | 15.63 | 1.47 | 0.24 | 2.67 |
| IR91477-79-1-1 | 25.33 | 20.00 | 1.32 | 0.36 | 2.33 |
| IR91477-81-1-1 | 44.69 | 21.67 | 1.30 | 0.27 | 3.00 |
| IR91477-124-1-1 | 45.45 | 18.49 | 1.93 | 0.45 | 1.67 |
| IR91477-125-1-1 | 54.50 | 19.13 | 1.64 | 0.38 | 2.33 |
| IR91477-136-1-1 | 34.39 | 15.80 | 1.53 | 0.35 | 3.00 |
| IR91477-137-1-1 | 51.88 | 22.12 | 3.38 | 0.68 | 1.00 |
| IR91477-148-1-1 | 47.09 | 23.31 | 2.04 | 0.51 | 1.00 |
| IR91477-150-1-1 | 60.83 | 27.63 | 2.53 | 0.59 | 1.00 |
| IR91477-167-1-1 | 38.08 | 21.00 | 1.83 | 0.48 | 1.67 |
| IR91477-170-1-1 | 34.33 | 20.83 | 1.60 | 0.37 | 3.67 |
| IR91477-196-1-1 | 47.11 | 18.61 | 1.34 | 0.31 | 2.33 |
| IR91477-250-1-1 | 37.66 | 17.67 | 1.08 | 0.23 | 3.00 |
| IR29 (sensitive check) | 19.00 | 10.30 | 0.63 | 0.13 | 8.00 |
| Hasawi (tolerant check) | 48.40 | 28.20 | 1.90 | 0.28 | 3.00 |
| Significance |  |  |  |  |  |
| Genotype | *** | *** | ** | * | *** |
| LSD (*P* <0.05) | 2.30 | 1.40 | 0.26 | 0.07 | 1.26 |
| CV (%) | 4.3 | 3.9 | 25.1 | 30.1 | 23.4 |
| Heritability (%) | 74.1 | 69.1 | 60.0 | 63.0 | 52.0 |

CV, coefficient of variation
